# A Central Test for Candidates

**Published:** 1920-10-16

**Authors:** 


### THE MATRONS' AND SISTERS' DEPARTMENT.

### A CENTRAL TEST FOR CANDIDATES.

The training-schools have not lost sight of the advice given them by Dr. Lane-Claypon to endeavour to attract probationers by making entrance less easy for them. In fact, the proposed entrants' examination, which at one time seemed a counsel of perfection, is beginning to take shape. It is universally admitted that the test cannot in the first instance be a very hard one. Indeed, the Junior Oxford or Cambridge has been suggested, which certainly does not err on the side of severity. But, matrons are asking, How will it work? How can the schools which are accepting girls they would not have looked at some six years ago pretend that they can refuse all who do not come up to even this not very exacting standard.

It may not, perhaps, be as difficult as it seems to get probationers to understand that they ought to show evidence of education before aspiring to be nurses. The first step must be for the more important training-schools in town and country to let it be known that they will give a preference to candidates who have passed an accepted test as a proof that they are ready to respond to the teaching of the school. The next step would be to let headmistresses know what educational requirement the candidates for nursing education should aspire to. This in itself, it is believed, would instantly cause the high schools to look with greater favour on nursing as a career for their scholars. It can hardly be conceived by any who will not take the trouble to see with others' eyes how contemptuous an attitude the training-schools appear to adopt towards the great educational centres by ignoring entirely the aims of all teachers, and regarding candidates merely from the point of view, so it might be inferred, of their bones and muscles. This pagan way of estimating a woman's fitness for becoming a nurse, all disregardful of the way she has used her time at school, is responsible for the veiled hostility perceptible in schoolmistresses when they allude to the nurses' career. Once fix a standard and all this will surely disappear.

There are a great many girls, eminently suitable to become nurses, who have either not had the opportunity of passing even the simplest examination or have missed it. What shall be done about these? There is a growing consensus of opinion that the College of Nursing might well enlarge its sphere to offer candidates the chance of making good before their applications go in to the training-schools they select. There is no doubt that the very fact of being offered preparation for a career has in it something alluring. Among the very large class of young women who are dissatisfied with their lives after leaving school because they want to do something for their fellows there are many who would respond to advertisements which should inform them that on a given date an examination would be held in certain subjects, preparatory to a course of special study for nursing candidates. Such a proposal would indicate that the hospitals expected something of their candidates, and were prepared in return to give much. It is easy to see that out of some such small beginning a regular course of preliminary training might be developed. For instance, would not the educational authorities in large towns be only too happy to arrange courses suitable for women who had proved their ability to take advantage of them by passing such an entrance examination as might be determined on? There is an immense deal of miscellaneous educational effort wasted in preparing students for no particular career. To seize the plastic years between school and the age of twenty and turn them to practical account by courses of physiology, domestic science, elementary chemistry, and the practical duties of the first-year probationer, would not reduce the term of training for the nurse, but it would abolish the probationer's worst trials, render her a useful assistant from the beginning, and lessen to an enormous extent the perplexities of the ward sister—shall we not say of the matron also? Many a true vocation might be saved if these initiatory difficulties were removed.

We do not believe that the preliminary school need be or ought to be residential. Such a course would be prohibitive as regards cost, and could never touch a tithe of the candidates required. An establishment for even twenty pupils would be large if all the pupils were to sleep on the premises, and there are hundreds of candidates needed in the time it would take to prepare twenty.

The pressing need is to make a beginning. Let the College once start an entrants' examination and we believe the rest would follow.

